# Robotic Pancreaticoduodenectomy in Elderly vs. Younger Patients: Systematic Review with Meta-Analysis

**DOI:** 10.3390/jcm15072744

**Published:** 2026-04-05

**Authors:** Dimosthenis Chrysikos, Nikolaos Taprantzis, Spiros Delis, Amir Shihada, Alexandros Samolis, Theodore Troupis

**Affiliations:** Department of Anatomy, Athens Medical School, National and Kapodistrian University of Athens, 11527 Athens, Greece; dixrys@yahoo.gr (D.C.); spirosgdelis@gmail.com (S.D.); ameer_shehade@hotmail.com (A.S.); asamolis@med.uoa.gr (A.S.); ttroupis@med.uoa.gr (T.T.)

**Keywords:** robotic pancreaticoduodenectomy, elderly, octogenarians, young patients, outcomes

## Abstract

**Background**: As life expectancy increases, more elderly patients require a pancreaticoduodenectomy (PD). While minimally invasive approaches are preferred, data indicating the safety of robotic PD in elderly patients remains limited. This study compares operative outcomes of robotic PD in elderly versus younger patients to define its oncological role. **Material and Methods**: A systematic search of PubMed, Embase, Web of Science, and Scopus identified studies comparing robotic pancreaticoduodenectomy in elderly versus younger patients. Robotic-exclusive cohorts were analyzed for perioperative outcomes, complications, and mortality. A meta-analysis was performed using R to calculate pooled prevalences, Odds Ratios (ORs) and Weighted Mean Differences (WMDs). **Results**: Elderly patients experienced significantly longer operative times (MD = 11.4 min) and hospital stays (MD = 7.76 days). They demonstrated higher odds of severe complications (Clavien–Dindo ≥ III: OR = 2.20), delayed gastric emptying (DGE) (OR = 2.34), and mortality (OR = 3.42). There were no significant differences in blood loss, transfusions, overall complications, pancreatic fistulae, bile leakage, hemorrhage, infection, readmission, or reoperation. Notably, age-stratified subgroup analyses revealed a distinct risk divergence: studies using an 80-year cutoff reported significantly higher odds of mortality and DGE, whereas 70-year-threshold studies demonstrated more pronounced odds for severe and overall complications. **Conclusions**: While robotic pancreaticoduodenectomy is feasible in elderly patients with comparable intraoperative blood loss and overall complication rates to younger patients, it does not eliminate all age-related risks. Elderly patients remain at significantly higher risk for severe complications and mortality. Therefore, robotic application in this demographic requires rigorous preoperative assessment, utilizing age as an initial risk-stratifier while allowing physiological reserve to determine final surgical candidacy.

## 1. Introduction

As global advancements in technological, medical, and socioeconomic sectors continue to drive an increase in human longevity, elderly patients have become a rapidly expanding demographic within surgical oncology [1]. Simultaneously, several studies have reported an association between increasing incidence of pancreatic malignancies and older age. This demographic shift will naturally lead to an increased demand for complex surgical interventions, making the establishment of evidence-based surgical management protocols essential [2].

Furthermore, variables like advanced age, potential adverse effects, and the frailty of elderly patients have significantly discouraged surgeons from operating on this vulnerable cohort [3,4]. However, recent technological advancements have provided medical professionals with the essential tools to perform safer operations [5,6]. Robotic operations play an expanding role in patient management in multiple different specialties. Studies and meta-analyses have shown promising results regarding robotic procedures in gastrointestinal, gynecological and other oncological cases [7,8]. Parallel to these technological advancements, the broader field of minimally invasive pancreatic surgery has also evolved to safely accommodate higher-risk populations.

Indeed, an increasing number of studies have shown positive results regarding laparoscopic pancreaticoduodenectomy (PD) in elderly patients, reporting lower numbers of complications and shorter recovery times [9,10]. Nevertheless, individual studies and diverse meta-analyses focused on minimally invasive techniques have reported conflicting data regarding the rate of severe complications and mortality in elderly patients compared to their younger counterparts [4,5,9,11,12]. A significant confounding factor in these previous analyses is that they frequently mixed laparoscopic and robotic PDs together. Because these modalities possess fundamentally different capabilities, as well as different demands, grouping them obscures the true independent impact of robotic technology on this vulnerable population.

Therefore, the primary novelty of this systematic review and meta-analysis lies in its exclusive isolation of robotic PD. By strictly excluding standard laparoscopic cases, this study aims to evaluate the safety and effectiveness of robotic PD in elderly patients through a direct, unconfounded comparison with younger counterparts, ultimately providing a clearer evidence-based framework for clinical decision-making.

## 2. Materials and Methods

We systematically searched through the PubMed, Embase, Web of Science and Scopus databases using the following key words: “Robotic pancreaticoduodenectomy” AND “elderly patients”, “robotic pancreaticoduodenectomy” AND “outcomes”, “elderly vs. younger patients”, “robotic pancreaticoduodenectomy” AND “octogenarians”, “robotic pancreaticoduodenectomy safety” AND “older vs. younger patients”, and “effectiveness of robotic pancreaticoduodenectomy” AND “elderly”, in order to collect data that would fit the criteria of our review. The search and study selection process followed the PRISMA guidelines [Supplementary File S1] [13]. Since this paper used only published data, neither institutional review board approval nor any other consent was needed.

The literature search was restricted to English-language publications. To ensure comprehensive coverage, the reference lists of relevant articles were manually screened to identify further eligible data. Ultimately, the inclusion of studies was determined by strict adherence to our predefined eligibility criteria, exclusively specifying studies that compared the outcomes of robotic pancreaticoduodenectomy between older and younger patients, or stratified the included patients based on a year cutoff above the age of 70. On the other hand, studies that included patients who also underwent a different type of PD, other than robotic, were not included.

Given the variation in age thresholds used to define elderly patients across the included studies, a subgroup analysis was conducted based on these specific cutoffs. Specifically, the included studies were stratified into two distinct groups: those utilizing a 70-year cutoff and those utilizing an 80-year cutoff.

Additionally, the collection of outcomes that were decided to be assessed and were reported by the literature included: operative blood loss (mL), operative time (min), postoperative transfusions, overall complications, severe complications, clinical pancreatic fistula, delayed gastric emptying, wound infection, mortality (within 90 days), length of stay (days), readmission (within 90 days), reoperation, bile leakage, and hemorrhage. Clinical pancreatic fistula, delayed gastric emptying, and hemorrhage were diagnosed in accordance with the International Study Group criteria [14,15,16,17]. Severe complications consist of complications of scale 3 and above according to the Clavien–Dindo classification [18].

Furthermore, studies involving animal models, unpublished conference abstracts, and publications with incomplete or unextractable data sets were systematically excluded from this analysis. We performed a meta-analysis using R (version 4.3.2) and RStudio, employing the “meta” and “metafor” packages for statistical analysis. To assess the comparative outcomes between older and younger patients, Odds Ratios (ORs) were calculated from study-level 2 × 2 contingency tables for dichotomous variables, while Weighted Mean Differences (WMDs) were used for continuous variables. For these comparative analyses, the Restricted Maximum Likelihood (REML) estimator was utilized to calculate the between-study variance (t^2^).

Where pooled prevalence estimates of intra- and postoperative outcomes were calculated for the elderly cohort alone (including mortality, complications, Clavien–Dindo III-IV complications, clinical pancreatic fistula, delayed gastric emptying, bile leakage, pancreatic hemorrhage, infection, intraoperative blood loss, operative time, transfusion, reoperation, and readmission), we employed the Freeman–Tukey double arcsine (PFT) transformation. The rationale for this transformation was to stabilize the variances of proportions, particularly for rare surgical complications with rates approaching 0 or 1, ensuring appropriate study weighting. For these prevalence estimates, the Hartung–Knapp (HK) estimator was applied.

Given the inherent clinical and methodological heterogeneity expected across observational surgical studies comparing varied patient populations, a random-effects model was chosen a priori and applied to all analyses. Heterogeneity was assessed using Cochran’s Q test and Higgins’ I^2^ statistic, interpreted as low (0–30%), moderate (30–50%), substantial (50–75%), or considerable (75–100%) heterogeneity. Statistical significance was defined as *p* < 0.05.

### 2.1. Data Extraction

Data retrieval was performed manually and independently by two investigators (D.C. and N.T.) without the assistance of automated extraction software. Information from the selected online databases was compiled into Microsoft Excel (version 16.98) spreadsheets, specifically capturing raw event counts and prevalence metrics. Following their separate extractions, the reviewers cross-examined their findings, addressing and resolving any data inconsistencies via mutual agreement.

### 2.2. Risk of Bias Assessment

The methodological quality and potential risk of bias of the included non-randomized observational studies were evaluated utilizing the Newcastle–Ottawa Scale (NOS) [19]. This appraisal was executed independently by two authors (D.C. and N.T.). Every NOS domain was individually scored to generate a cumulative risk profile, categorized as low, moderate, or high, for each article. Any diverging evaluations between the two reviewers were reconciled through detailed deliberation to achieve consensus [Table S1]. In order to assess the risk of publication bias, funnel plots were created for the effect sizes of each variable. As there were not enough studies to fully conduct Egger’s tests for all variables, a qualitative assessment of funnel plots was performed [Supplementary File S2].

## 3. Results

**Figure 1:**
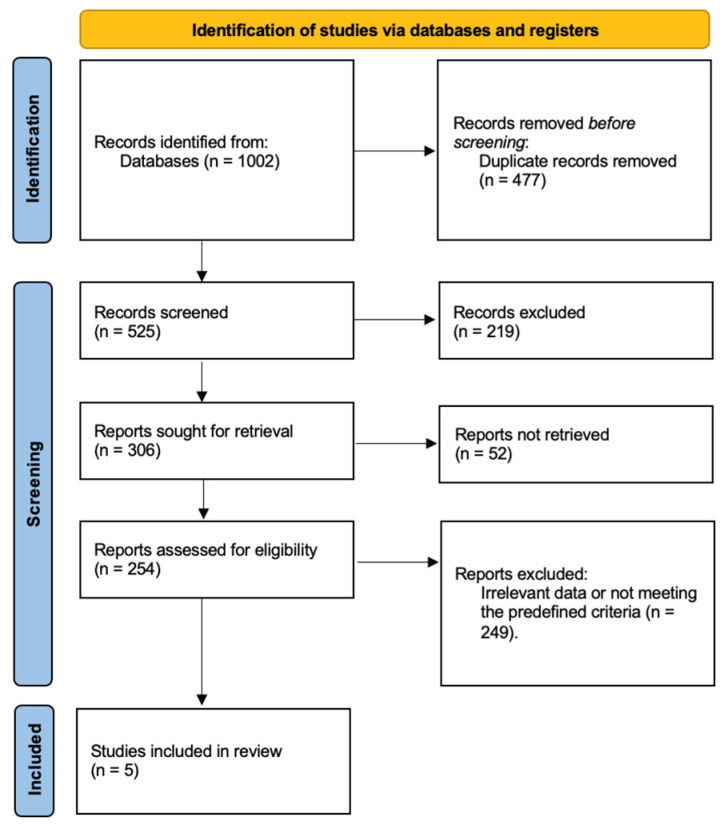
PRISMA flow chart for the study selection process.

After the completion of our systematic search, 1002 studies were identified from the database and citation searches. Following the removal of duplicates and articles that did not meet our predefined criteria, 254 studies were assessed for eligibility. Finally, it was decided that five studies would be included in the systematic review [Figure 1].

Among the included studies, 281.0 individuals was the mean sample per study, with 48.4 for the elderly and 232.6 for the younger patients. Three studies put their age cutoff at 70 years, while the remaining two put it at 80 years. The study characteristics are presented in Table 1.

### 3.1. Blood Loss and Transfusion Rate

The mean estimated blood loss during robotic PD in the younger cohort was 196.6 mL and 233.2 mL in the older group. Notably, the mean difference between the two was not found to be significant (MD = 28.0; 95% CI: −1.33, 57.38). The age subgroup analysis also showed no significant difference between the studies with a different age cutoff [Supplementary Table S3].

**Figure 2:**
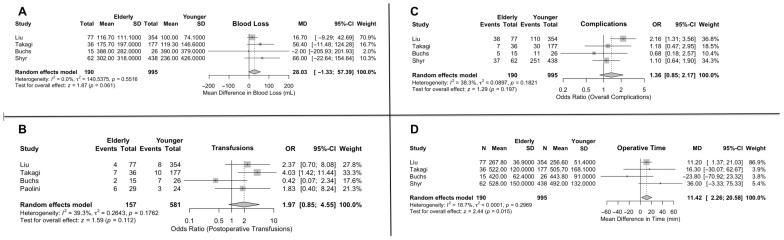
Outcomes of robotic pancreaticoduodenectomy in elderly vs. younger patients. (**A**) Blood loss. (**B**) Transfusions. (**C**) Overall complications. (**D**) Operative time.

As far as the postoperative transfusions are concerned, 13.5% of the older cohort received blood transfusions, while only 8.1% of the younger patients did. The effect size between the two was not, however, significant (OR = 1.97; 95% CI: 0.85, 4.55) [Figure 2]. The age subgroup analysis also showed no significant difference between the studies with a different age cutoff [Supplementary Table S2].

### 3.2. Total Operative Time

Regarding the total operative time, the mean estimated time of surgery for the older cohort was 433.4 min and 424.1 min for the younger individuals. The MD between the two values was found to be significant, meaning that the older cohort underwent notably longer operations (MD = 11.4; 95% CI: 2.26, 20.58) [Figure 2]. The age subgroup analysis showed no significant difference in the effect size between the studies with a different age cutoff [Supplementary Table S3].

### 3.3. Overall Complications

The mean estimated prevalence of postoperative complications was 40.8% for the older patients and 35.3% for the younger cohort. The effect size for all postoperative complications between the older and younger patients was OR = 1.36; 95% CI: 0.85, 2.17. The difference between the two groups, however, was not statistically significant [Figure 2]. Subgroup analysis showed that the OR in the studies with a 70-year cutoff was significantly greater than the effect size in the 80-year cutoff group (OR = 1.95; 95% CI: 1.27, 3.01 and OR = 0.88; 95% CI: 0.52, 1.52, respectively, with a *p*-value = 0.0091) [Supplementary Table S2].

### 3.4. Clinically Severe Complications

**Figure 3:**
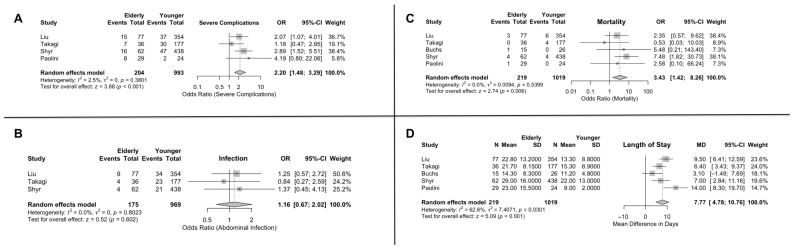
Outcomes of robotic pancreaticoduodenectomy in elderly vs. younger patients. (**A**) Severe complications. (**B**) Infection. (**C**) Mortality. (**D**) Length of stay.

Clavien–Dindo III–IV complications occurred in 20.7% of older patients compared to 10.4% in the younger group. Interestingly, the effect size for particularly severe complications (Clavien–Dindo III–IV) was found to be significant, indicating higher odds of severe adverse outcomes in the elderly patients (OR = 2.20; 95% CI: 1.47, 3.28) [Figure 3]. Subgroup analysis showed that the OR in the studies with a 70-year cutoff was significantly greater than the effect size in the 80-year cutoff group (OR = 2.44; 95% CI: 1.02, 5.83 and OR = 2.00; 95% CI: 0.94, 4.26, respectively, with a *p*-value = 0.0265) [Supplementary Table S2].

### 3.5. Infection

As far as wound infection is concerned, the mean estimated prevalence was 10.1% for older patients and 8.5% for the younger patients. The included studies did not show a significant difference between the two groups, with the effect size being OR = 1.15; 95% CI: 0.66, 2.01 [Figure 3]. The age subgroup analysis also showed no significant difference between the studies with a different age cutoff [Supplementary Table S2].

### 3.6. Mortality

The estimated pooled mortality was 3.26% for the older individuals and 0.76% for the younger individuals. The overall effect size between the two patient cohorts was found to be significant, as the elderly patients were found to have higher mortality odds following robotic PD (OR = 3.42; 95% CI: 1.42, 8.26) [Figure 3]. Subgroup analysis showed that the OR in the studies with an 80-year cutoff was significantly greater than the effect size in the 70-year cutoff group (OR = 4.68; 95% CI: 0.71, 8.10 and OR = 2.39; 95% CI: 1.31, 16.78, with a *p*-value = 0.0223) [Supplementary Table S2].

### 3.7. Postoperative Length of Stay

The estimated mean length of stay was 22.1 days for the elderly cohort and 14.1 days for the younger cohort. This variable was found to be significantly higher in the elderly patients than in the younger patients, with an MD = 7.76; 95% CI: 4.77, 10.76 [Figure 3]. Subgroup analysis showed that the MD in the studies with a 70-year cutoff was significantly greater than the effect size in the 80-year cutoff group (MD = 8.66; 95% CI: 4.09, 13.23 and MD = 6.68; 95% CI: 1.40, 11.95, respectively, with a *p*-value < 0.0001) [Supplementary Table S3].

### 3.8. Readmission

**Figure 4:**
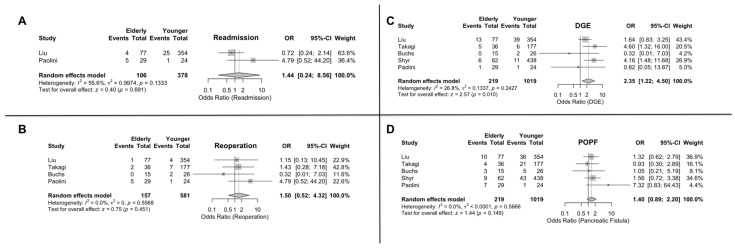
Operative outcomes of robotic pancreaticoduodenectomy in elderly vs. younger patients. (**A**) Readmission. (**B**) Reoperation. (**C**) Delayed gastric emptying. (**D**) Clinical pancreatic fistula.

The estimated prevalence of readmission was 9.7% for the elderly patients and 6.9% for the younger patients. The effect size was not found to be significantly different between the two groups (OR = 1.43; 95% CI: 0.24, 8.56) [Figure 4]. Subgroup analysis was not feasible for this variable.

### 3.9. Reoperation

The estimated prevalence of reoperation was 4.2% for the elderly patients and 2.5% for the younger patients. Similar to readmission, the effect size was not significantly different between younger and older patients, with an effect size OR = 1.50; 95% CI: 0.52, 4.31 [Figure 4]. The age subgroup analysis also showed no significant difference between the studies with a different age cutoff [Supplementary Table S2].

### 3.10. Clinical Pancreatic Fistula

The estimated prevalence of clinical pancreatic fistula was 15.4% for the older cohort and 10.5% for the younger group. The effect size for clinical pancreatic fistula (PF) was OR = 1.39; 95% CI: 0.88, 2.20. This outcome was not, however, significantly different between the two groups [Figure 4]. The age subgroup analysis also showed no significant difference between the studies with a different age cutoff [Supplementary Table S2].

### 3.11. Delayed Gastric Emptying

The estimated prevalence of delayed gastric emptying (DGE) was 12.4% in the elderly patients and 5.1% in the younger patients. The difference between postoperative DGE results was significant between the two cohorts, with the effect size being OR = 2.34; 95% CI: 1.22, 4.50. Thus, the older patients had a higher odd of experiencing such complications [Figure 4]. Subgroup analysis showed that the OR in the studies with an 80-year cutoff was significantly greater than the effect size in the 70-year cutoff group (OR = 4.33; 95% CI: 1.96, 9.60 and OR = 1.49; 95% CI: 0.78, 2.85, respectively, with a *p*-value < 0.0001) [Supplementary Table S2].

### 3.12. Bile Leakage

**Figure 5:**
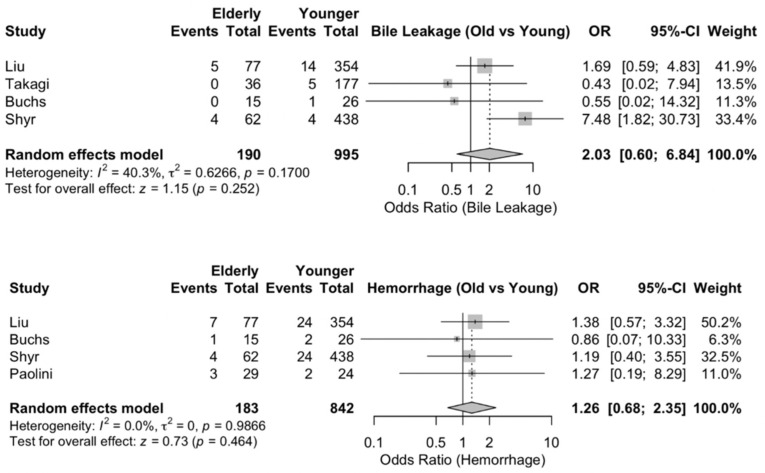
Operative outcomes for robotic pancreaticoduodenectomy in elderly vs. younger patients. (**Top**) Bile leakage. (**Bottom**) Hemorrhage.

The estimated prevalence of bile leakage was 3.0% in the elderly group and 2.1% in the younger group. The effect size for postoperative bile leakage was OR = 2.03; 95% CI: 0.60, 6.83. The difference between the two patient cohorts, however, was not found to be significantly different [Figure 5]. The age subgroup analysis also showed no significant difference between the studies with a different age cutoff [Supplementary Table S2].

### 3.13. Hemorrhage

The estimated prevalence of hemorrhage complications was 7.7% in the older cohort and 5.4% in the younger group. The effect size for postoperative hemorrhage was OR = 1.26; 95% CI: 0.67, 2.35. This outcome, however, was not statistically different between old and young patients [Figure 5]. The age subgroup analysis also showed no significant difference between the studies with a different age cutoff [Supplementary Table S2].

The detailed operative outcomes are presented in Table 2 and Table 3. The age subgroup analysis is presented in Supplementary File S1.

### 3.14. Publication Bias Assessment

A qualitative visual assessment of the funnel plot for each outcome was conducted. No gross asymmetry, indicative of publication bias, was identified. However, the limited number of included studies restricts the confidence in our interpretation [Supplementary File S2].

## 4. Discussion

The clinical decision to undergo surgery in distinct patients group relies on a variety of factors that contribute to the postoperative outcomes and subsequent clinical trajectory of each individual. The elderly patient population consists of a significantly interesting group that requires special management and evaluation when surgical operation is recommended. As technology evolves, more advanced techniques are developed that improve on the shortcomings of previously utilized instruments and methods [25,26,27]. Thus, when compared to other available options, existing reviews have shown safety advantages of robotic pancreaticoduodenectomy in the general population regarding postoperative complications, blood loss, wound infection, etc. [28,29,30]. Other analyses have focused on elderly patients and their postoperative course following minimally invasive procedures [11]. However, in these studies, robotic PD was heavily underrepresented, resulting in uncertain comparisons and results.

Regardless of statistical significance, all of our results indicate a potential association between older age and higher unwanted operative outcomes.

The strongest, and most important, significant connection that was reported in this analysis was the increased mortality risk in the elderly patients compared to the younger patients (OR = 3.42). Kamarajah et al. reported a total mortality for robotic PD of 2%, which falls between the mortality rates of our two patient groups [6]. While the majority of evaluated outcomes showed no statistical variation between groups, the elderly cohort faced a substantially higher mortality risk. This observation aligns with existing evidence from the meta-analyses of Ballarin et al. and Zhu et al., which similarly reported increased mortality in older patients undergoing minimally invasive PD, the vast majority of which were performed laparoscopically [11,12]. Even though the individual mortality prevalences for each group were decreased with robotic PD, the difference in odds between the old and the young was bigger in our analysis (OR = 3.42 vs. OR = 2.61), indicating that while the robotic platform may lower the general risk, it appears unable to overcome the physiological vulnerabilities of elderly individuals, thereby widening the relative safety gap between age groups. Importantly, although the primary studies were conducted at high-volume, specialized centers by highly experienced robotic surgical teams, the inability to statistically adjust for unmeasured confounders, such as baseline comorbidities, frailty, or tumor stage, precludes definitive causal interpretations. Consequently, rather than strictly attributing this higher mortality to procedural or institutional factors, we suggest that this disparity may be associated with intrinsic age-related vulnerabilities that leave older patients more susceptible to adverse operative outcomes.

Additionally, our subgroup analysis evaluating the mortality effect size across different age cutoffs demonstrated a crucial inflection point for surgical decision-making. Specifically, the data suggests that an age threshold of 80 years represents a critical boundary where the risk of postoperative mortality increases substantially. While patients in their 70s demonstrate a relatively lower and more acceptable mortality risk profile, the markedly elevated vulnerability among octogenarians dictates a far more cautious approach. Consequently, an age of 80 or above should serve as a major clinical trigger for rigorous comprehensive geriatric assessment, frailty screening, and highly individualized shared decision-making before offering robotic pancreaticoduodenectomy.

Regarding overall morbidity, our analysis yielded a non-significant OR of 1.36 and a 40.8% complication rate in elderly patients, representing a marginal improvement over the OR of 1.45 and 47% prevalence reported by Ballarin et al. [11]. While these findings do not allow us to reach any absolute conclusions, the potential for robotic PD to manage overall complications with more efficiency in both age groups can be seen. However, the association between serious complications and older patients is much stronger in our study (Clavien–Dindo III-IV OR = 2.20, 20.7% vs. 10.4%). The existing literature contains conflicting reports concerning this outcome. Ballarin et al. reported a statistically different OR of 1.57, while Zhu et al. reported a nonsignificant OR of 1.40 [11,12]. Similarly, most studies that assessed serious complications in elderly patients after laparoscopic PD showed no significant differences: Wang et al. reported an OR = 1.55, Zhang et al. an OR = 1.43 (*p*-value = 0.02), and Bartos et al. an RR = 1.45 [10,31,32]. Based on these comparisons, there is a potential association between robotic PD and greater differences in postoperative morbidities in elderly and young patients that is not observed for other operative techniques. This significantly higher odds of serious complications could also partially explain the mortality rate differences that we discussed earlier. Despite the fact that the comparison with the existing literature showed that robotic PD is associated with a lower overall pooled prevalence of serious complications in non-elderly patients (10.4% vs. 11.0%/15.5%), the “gap” between older and younger patient safety seems to become greater with this operative method. This suggests that the profound physiological vulnerability of elderly individuals may limit the extent to which the technical benefits of robotic PD can translate into a reduction of severe morbidity. Consequently, while the robotic approach successfully minimizes surgical trauma, serious complications remain a persistent threat for the older population, possibly influenced by their baseline physiological status. Our subgroup analysis regarding overall and severe morbidity highlights a critical divergence in the timeline of age-related surgical risk. The effect size for overall and severe complications was notably greater in studies that utilized a 70-year cutoff compared to those that utilized an 80-year cutoff. This suggests that the physiological risk for developing postoperative complications increases substantially at an earlier age compared to the risk of mortality. Specifically, the data indicates that the physiological transition from the sixth to the seventh decade of life represents a steep escalation in morbidity risk. Conversely, as patients in their 70s already possess an elevated baseline risk for complications, the relative increase in morbidity when transitioning into the 80s is less pronounced. Therefore, transition into the seventh decade could be associated with a significant increase in surgical morbidity, even if the absolute mortality risk does not really augment until age 80.

Furthermore, delayed gastric emptying was the last variable in the first category of outcomes that was significantly different between the two patient cohorts (OR = 2.34). The majority of the existing literature does not report any changes in delayed gastric emptying outcomes between older and younger patients undergoing mainly laparoscopic PD [11,12,31]. The bigger difference in DGE was observed in the Takagi et al. study, which included the highest mean age for the elderly group out of all the assessed studies. This could potentially be an indicator that abdominal function increasingly deteriorates as the age of patients exceeds a certain limit [21].

Regarding the second set of operative outcomes, significant results were observed in the operative time and length of stay domains. The comparable operative blood loss observed between the two groups highlights the potential of the robotic platform to minimize intraoperative losses and maximize surgical efficiency. On the other hand, elderly patients underwent surgical operations that lasted significantly longer, indicating the complexity and increased degree of difficulty. Additionally, the markedly longer hospitalization periods observed in the older cohort support the notion that the physiological benefits of robotic PD do not appear to be able to mitigate the effects of advanced age. Despite the reduced surgical trauma afforded by this technique, the baseline frailty of elderly patients necessitates more extensive recovery windows. The prolonged operative time, the longer period of hospital stays, as well as similar blood losses were all supported by the minimally invasive PD meta-analyses [11,12].

As far as the subgroup analyses results for delayed gastric emptying and postoperative length of stay are concerned, the same logic applied to overall complications holds true. The risk for these outcomes increases significantly with the start of the seventh decade but does not proportionally augment when extending into the 80s. This reinforces the notion that the primary decline in gastrointestinal function and overall recovery becomes more evident after the 70th year, establishing a baseline morbidity risk that plateaus for octogenarians.

Overall, our findings support the notion that robotic PD is associated with lower rates for the majority of operative outcomes in older patients, the numbers being similar to those recorded for younger individuals. To a large extent, the robotic platform appears to mitigate age-associated risks related to immediate surgical trauma, as evidenced by comparable blood loss and minor complication rates. The main differences that were observed were mainly related to more clinically severe consequences, where the advanced abilities of the robotic PD could not fully overcome the inherent frailty of the elderly patients. In other words, even though the robotic method was associated with relatively low severe morbidities in the younger group, the physiological limits of older patients likely play an impactful role in their clinical trajectory, resulting in a persistently higher rate of severe morbidity and mortality despite the use of a highly advanced, minimally invasive platform. Finally, our subgroup analyses reveal a distinct timeline for this physiological vulnerability: while the transition into the 70s serves as the primary inflection point for increased surgical morbidity and delayed functional recovery, reaching the age of 80 marks a critical threshold for postoperative mortality. Therefore, an age-specific preoperative risk stratification is essential to maximize efficacy and minimize unwanted complications.

A critical factor when interpreting these findings is the inherent selection bias present in the primary literature. A review of the inclusion criteria from each study confirms that the elderly cohorts do not entirely represent the average aging population but rather a highly selected, relatively healthy subgroup of senior individuals. For instance, primary studies explicitly required elderly candidates to demonstrate independent mobility and activities of daily living or systematically excluded those with serious cardiopulmonary and hepatorenal insufficiencies [20,21,22,23,24]. Therefore, when translating these findings into real-world clinical practice, it is crucial to recognize that the safety and efficacy of robotic PD demonstrated in this analysis apply specifically to a rigorously chosen demographic. These results underscore the necessity of strict preoperative screening, confirming that robotic PD is a viable option for elderly patients, provided they meet physiological criteria similar to those utilized in these studies. Based on these synthesized findings, robotic pancreaticoduodenectomy should not be blindly considered for all elderly patients. Rather, its clinical application must be governed not only by chronological age alone, but by the physiological reserve of the patients as well. We propose a two-tiered approach to preoperative evaluation. First, chronological age may serve as an initial risk-stratification trigger: patients in their 70s warrant comprehensive counseling regarding the increased potential for severe morbidity and prolonged recovery, while octogenarians should be evaluated with careful consideration of their notably higher mortality risk. Second, physiological reserve, rather than chronological age alone, should ultimately guide final surgical eligibility. Ultimately, while the robotic platform minimizes operative trauma, it cannot reverse biological frailty; therefore, meticulous preoperative frailty screening and shared decision-making play an important role in safely bridging the gap between surgical technology and geriatric physiology.

### 4.1. Strengths

This analysis is strengthened by a clearly defined and independently executed study selection and data extraction process that can reduce the risk of bias. A clinical strength of this study is the high comparability of surgical expertise across the included studies, as all primary cohorts were operated on by high-volume, dedicated minimally invasive teams or at specialized mentor centers. Thus, our pooled estimates are not limited by a steep learning curve that is traditionally associated with robotic pancreaticoduodenectomy. Moreover, the analytical rigor of this review is reinforced by our comprehensive statistical approach, which encompasses the calculation of aggregate prevalences and comparative effect sizes, visual publication bias evaluations via funnel plots, and systematic quality appraisals using the Newcastle–Ottawa Scale. Ultimately, our commitment to methodological transparency, demonstrated by the application of open-source software and standardized analytical frameworks, ensures the high reproducibility and dependability of our synthesized conclusion. Furthermore, by implementing novel age-stratified subgroup analyses, we addressed any inherent clinical heterogeneity introduced by differing age cutoffs. Finally, the overall heterogeneity remained low throughout the majority of the studies, indicating a consistency in results and highlighting the significance of combining them in a meta-analysis.

### 4.2. Limitations

This systematic review and meta-analysis presented has a few limitations. Primarily, the inclusion of only five studies inherently limits the overall sample size and the statistical power of our pooled analyses. This restricts the precision of our between-study heterogeneity estimates, inevitably resulting in wider confidence intervals, and impacts the overall robustness of the conclusions. All included primary studies are retrospective and observational in nature. Consequently, they carry an inherent high risk of unmeasured confounding bias, as the observed outcomes may be influenced by variables not fully captured or adjusted for in the primary data. Specifically, we were unable to mathematically adjust our pooled estimates for critical patient and oncological confounders, including baseline comorbidities, specific frailty indices, tumor stages, and American Society of Anesthesiologists (ASA) scores. Since the observed outcomes may be influenced by these baseline cohort disparities rather than the surgical modality alone, the reader needs to interpret the results carefully. Furthermore, the age cutoff was not entirely consistent across all studies, as it ranged from 70 to 80 years old. Even though subgroup analyses were conducted to counter this limitation, the reader needs to interpret the results carefully, while taking into consideration the aforementioned variability and the small sample sizes within these specific subgroups. In other words, the risk of classification bias could potentially dilute the comparative effect sizes, narrowing the true disparity between strictly defined age groups. Additionally, the geographic concentration of the included primary literature restricts the external validity of our findings, while also potentially contributing to heterogeneity in our findings.

The limited number of included studies did not allow us to conduct any statistical tests (Egger’s tests) to evaluate the risk of publication bias, or any sensitivity analysis to evaluate the individual influence of each included study. Although qualitative visual inspection of funnel plots revealed no gross asymmetry, undetected publication bias may still exist due to the limited number of assessed studies. If present, this could potentially affect the analysis’s results, thereby restricting broader generalizability. There were some limitations to clinical data reporting, as not all studies reported the same outcomes, e.g., readmission. Furthermore, evaluating long-term oncological outcomes (overall survival and recurrence) was unfeasible due to inconsistent reporting and heterogeneous follow-up assessments across the primary studies. While this limits our conclusions and certainty regarding long-term survival, the short-term variables evaluated in this study remain highly significant clinical parameters that should assist in initial preoperative risk stratification for patients and surgical decision-making. Thus, larger, multicenter prospective studies are needed in order to obtain a clearer picture and more reliable conclusions regarding the role of robotic PD in the elderly population.

## 5. Conclusions

Robotic PD seems to be an effective and safe surgical procedure across different ages, as the majority of overall complication rates were fairly similar between the two groups. However, the significant differences in severe complications and mortality suggest that the frailty of elderly patients supersedes the technical advantages of robotic PD. While the robotic method limits the overall risk of the surgical process for both cohorts, the younger population appears to benefit more, making the safety “gap” between the two wider. Importantly, our findings also support the idea that this vulnerability occurs in phases, as morbidity escalates at around age 70, while mortality risk is significantly increased in patients entering their eighth decade.

## Figures and Tables

**Table 1:** Detailed characteristics of the included studies.

Studies	Year	Area	Age	Total Patients	Risk of Bias Assessment
Liu [20]	2020	Asia	Elderly: 77.0	77	Low Risk
			Younger: 57.9	354	
Takagi [21]	2025	Asia	Elderly: 82.7	59	Low Risk
			Younger: 68.0	321	
Buchs [22]	2010	America	Elderly: 76.8	15	Low Risk
			Younger: 56.3	26	
Shyr [23]	2023	Asia	Elderly: 85.0	62	Low Risk
			Younger: 63.0	438	
Paolini [24]	2021	Europe	Elderly > 70	29	Low Risk
			Younger < 70	24	

**Table 2:** Pooled outcomes of robotic pancreaticoduodenectomy in the old vs. young patients.

Parameters	Odds Ratio	Prevalence in Older Patients (%)	Prevalence in Younger Patients (%)
Transfusions	1.97	13.5	8.1
Complications	1.36	40.8	35.3
Mortality	3.42	3.26	0.76
Clavien–Dindo III-IV	2.20	20.7	10.4
Infection	1.15	10.1	8.5
Reoperation	1.50	4.2	2.5
Readmission	1.43	9.7	6.9
Bile Leakage	2.03	3.0	2.1
Clinical Pancreatic Fistula	1.39	14.5	10.5
Hemorrhage	1.26	7.7	5.4
Delayed Gastric Emptying	2.34	12.4	5.1

**Table 3:** Peri- and postoperative measurement values for the elderly and younger patients.

Parameters	Mean Difference	*p*-Value	Pooled Estimates in Older Patients	Pooled Estimates in Younger Patients (%)
Operative Time	11.4 min	0.0145	433,4 min	424.1 min
Blood Loss	28.0 mL	0.0614	233.2 mL	196.6 mL
Length of Stay	7.76 days	<0.0001	22.1 days	14.1 days

## Data Availability

No new data were created or analyzed in this study. Data sharing is not applicable to this article.

## Supplementary Materials

The following supporting information can be downloaded at https://www.mdpi.com/article/10.3390/jcm15072744/s1: File S1: PRISMA checklist; File S2: Publication Bias Qualitative Assessment; Table S1: Risk of bias assessment; Table S2: Age cutoff subgroup analysis for OR effect size; Table S3: Age cutoff subgroup analysis for MD effect size.

## Abbreviations

The following abbreviations are used in this manuscript:

PFT	Freeman–Tukey Double Arcsine
DL	DerSimonian–Laird
NOS	Newcastle–Ottawa Scale
DGE	Delayed Gastric Emptying
PF	Pancreatic Fistula
PD	Pancreaticoduodenectomy
OR	Odds Ratio
MD	Mean Difference
