# Supplementary material for: Robotic Pancreaticoduodenectomy in Elderly vs. Younger Patients: Systematic Review with Meta-Analysis
Source: J Clin Med. 2026 Apr 5;15(7):2744. doi: 10.3390/jcm15072744 (PMC13072716; doi:10.3390/jcm15072744)

Publication Bias Qualitative Assessment

After a careful visual assessment of all funnel plots, no gross asymmetry, indicative of publication bias, was detected. Despite this, the limited number of studies restricts the confidence of our interpretation.

Figure S1: Funnel plot for blood loss

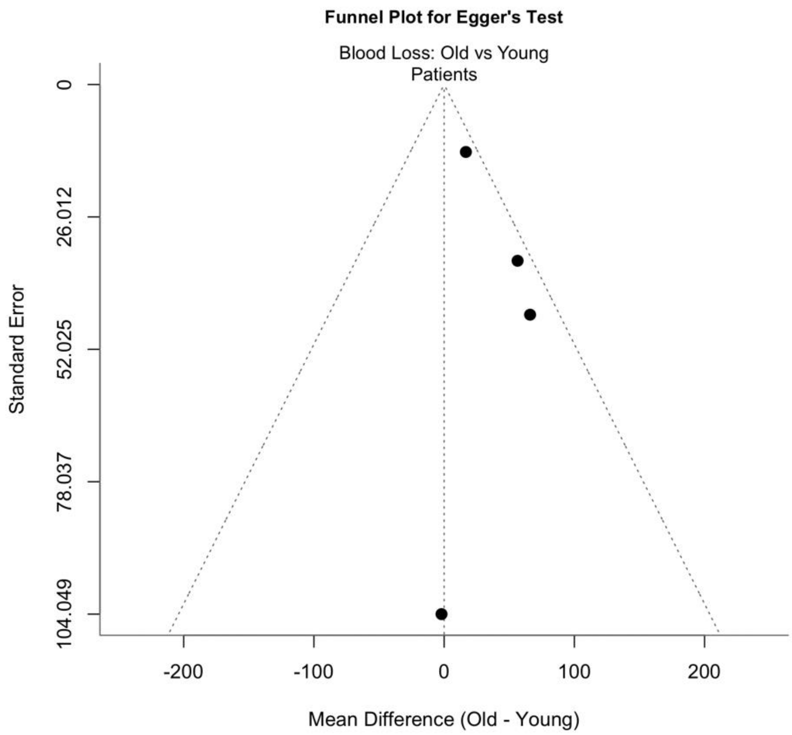

Figure S2: Funnel plot for transfusion

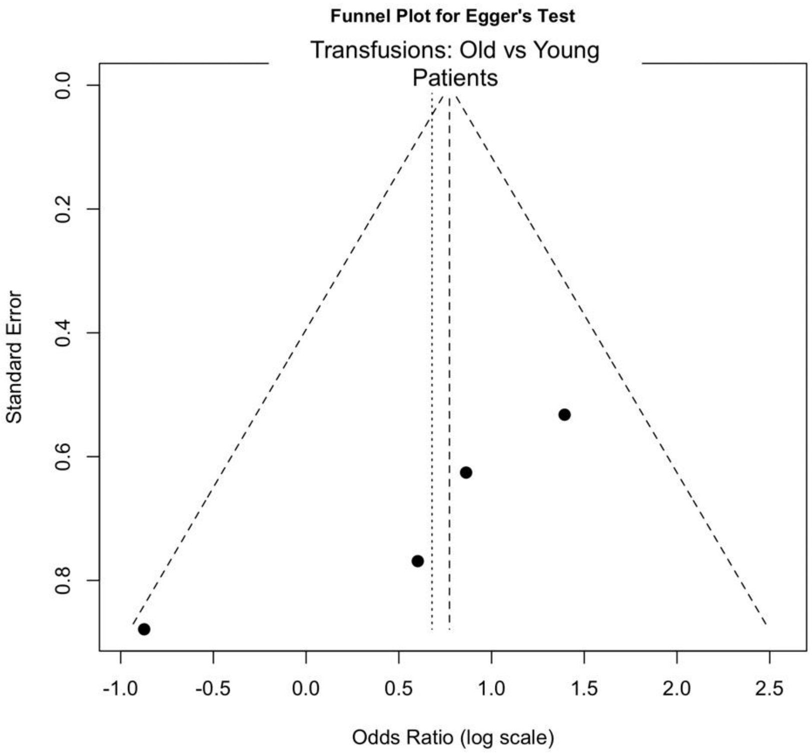

Figure S3: Funnel plot for operative time

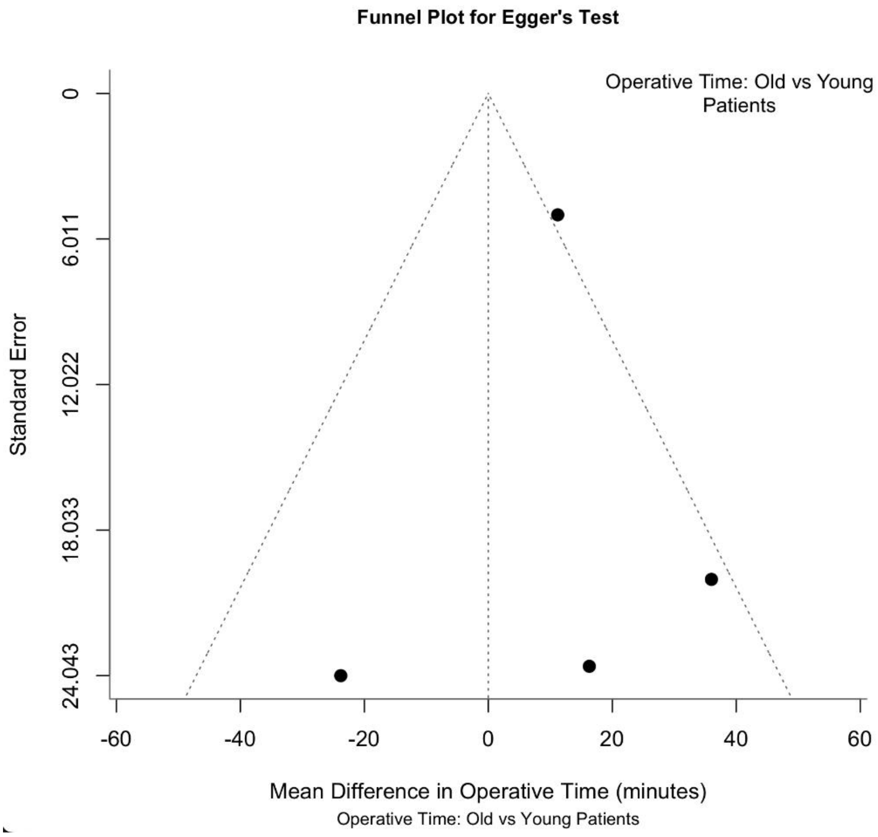

Figure S4: Funnel Plot for total complications

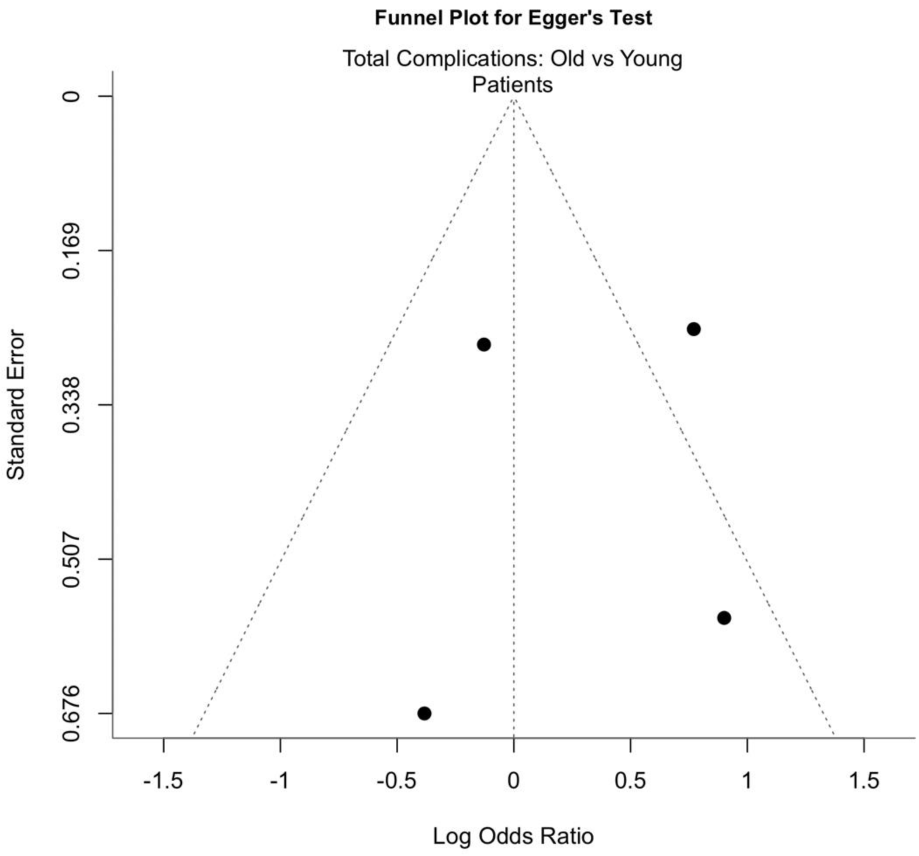

Figure S5: Funnel Plot for severe complications

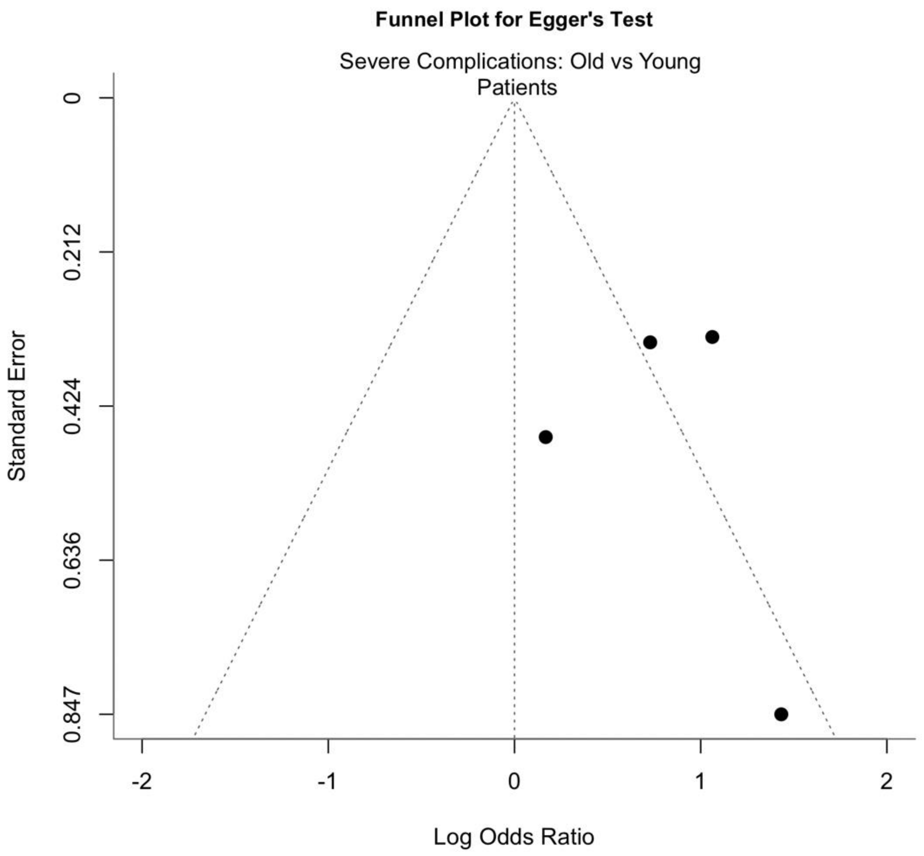

Figure S6: Funnel plot for wound infection

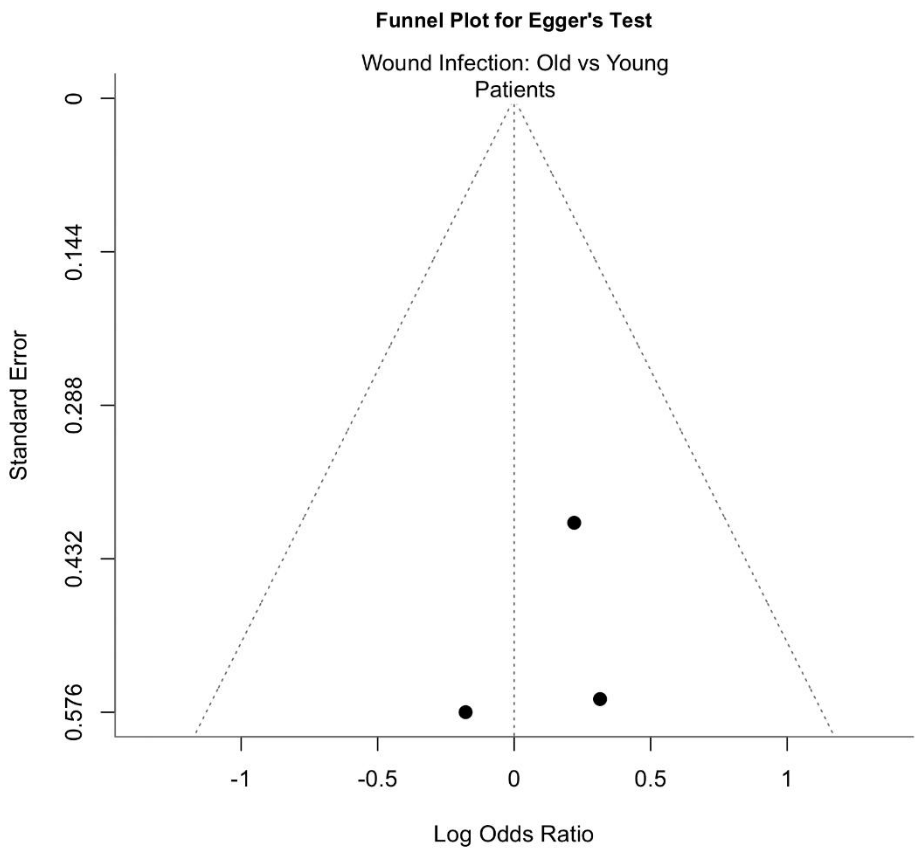

Figure S7: Funnel plot for mortality

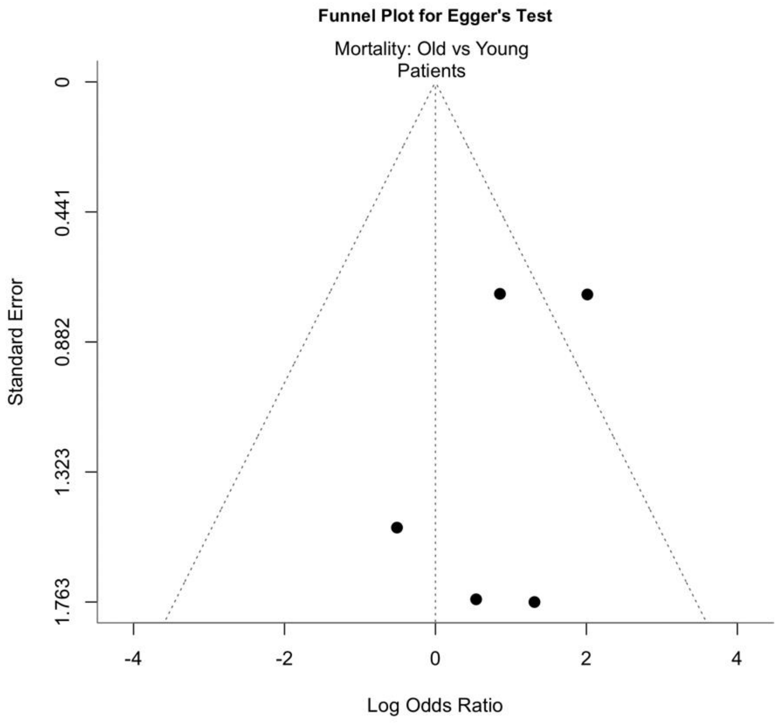

Figure S8: Funnel plot for length of stay

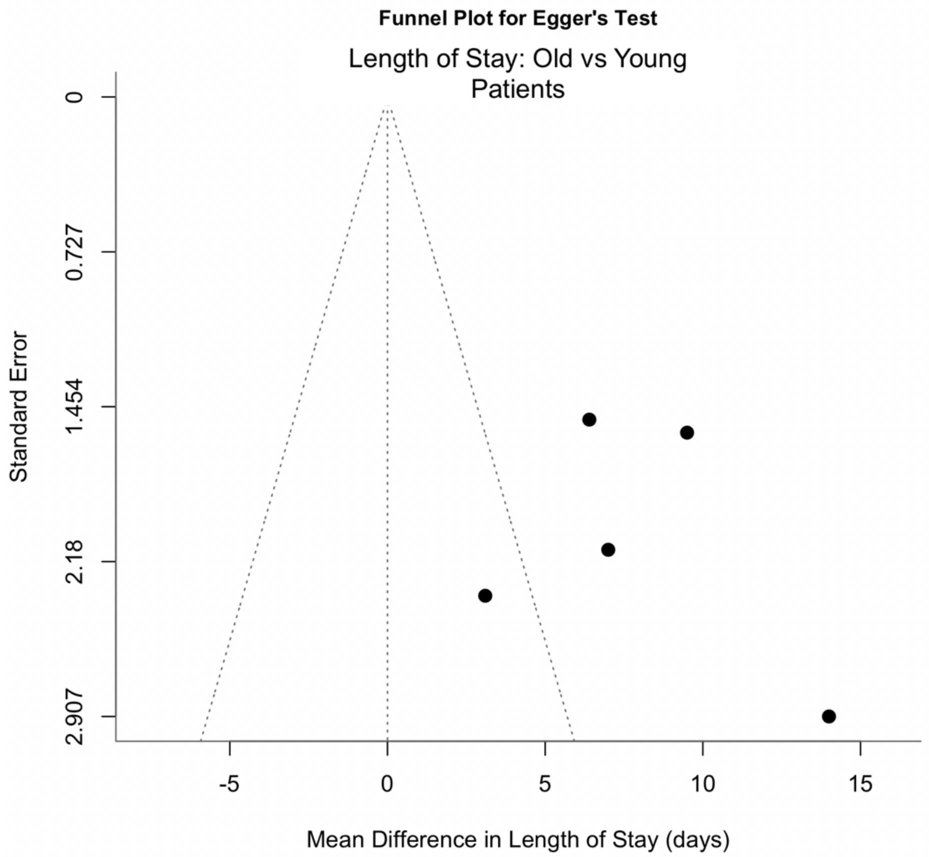

Figure S9: Funnel plot for reoperation

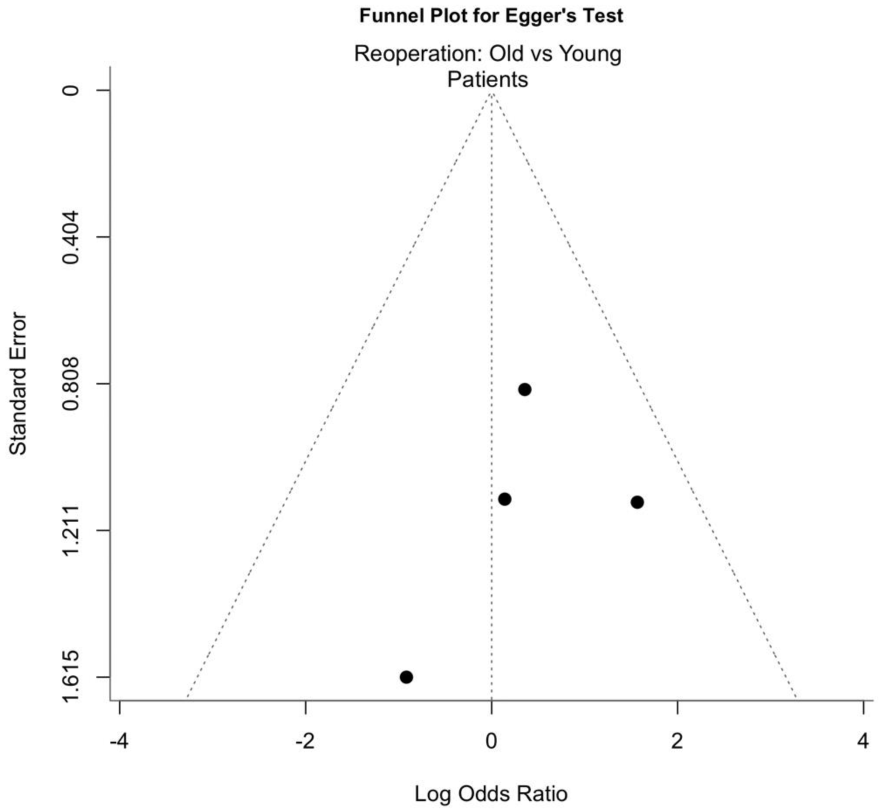

Figure S10: Funnel plot for pancreatic fistula

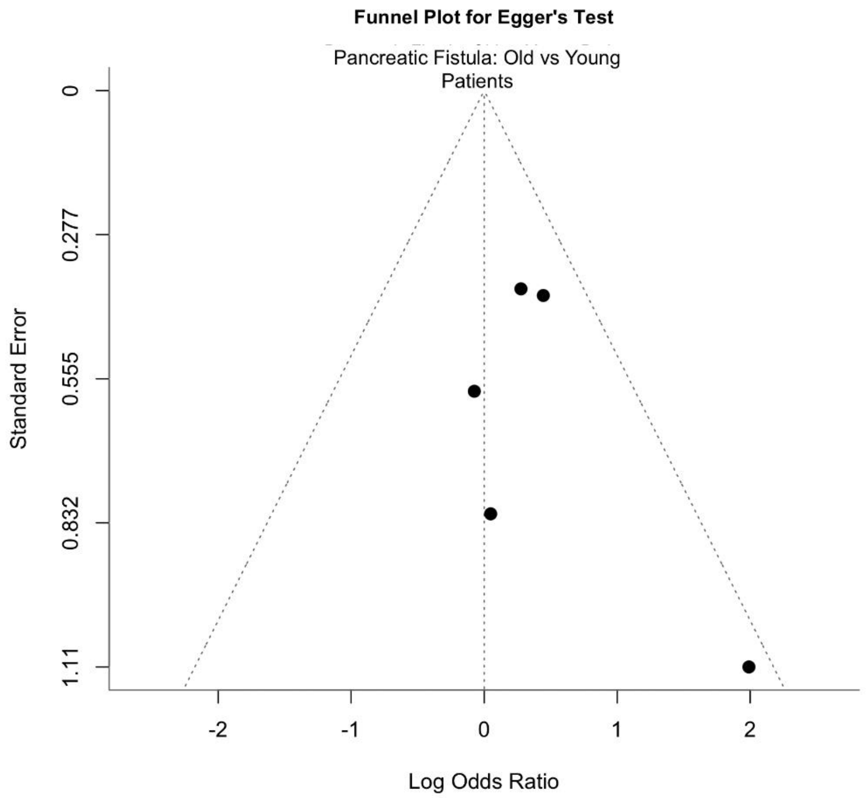

Figure S11: Funnel plot for delayed gastric emptying

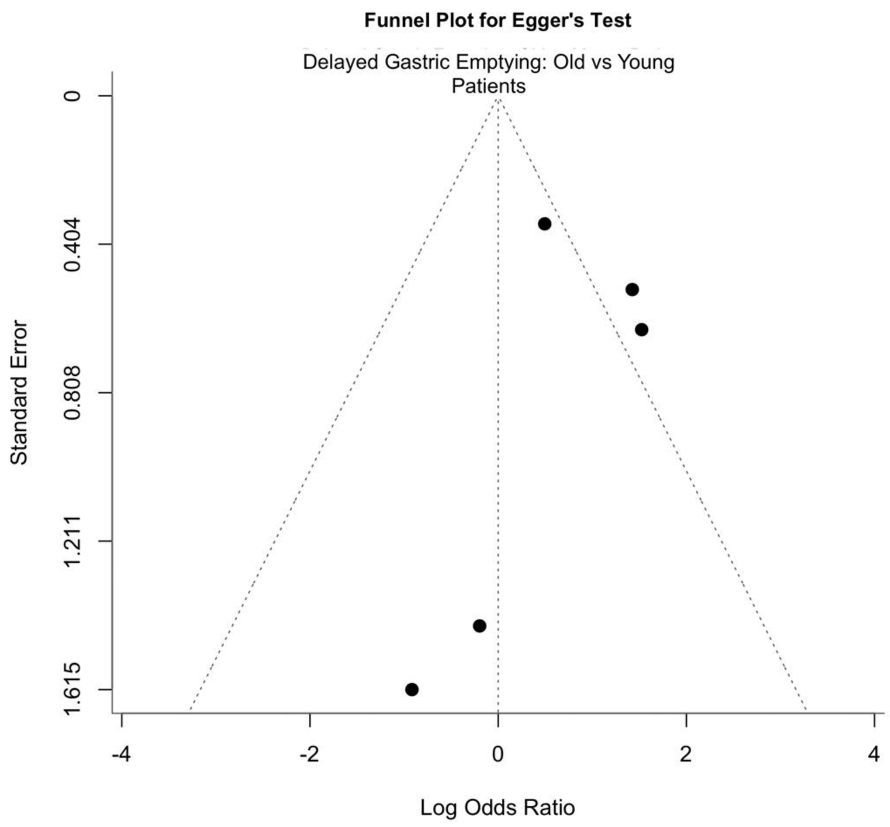

Figure S12: Funnel plot for bile leakage

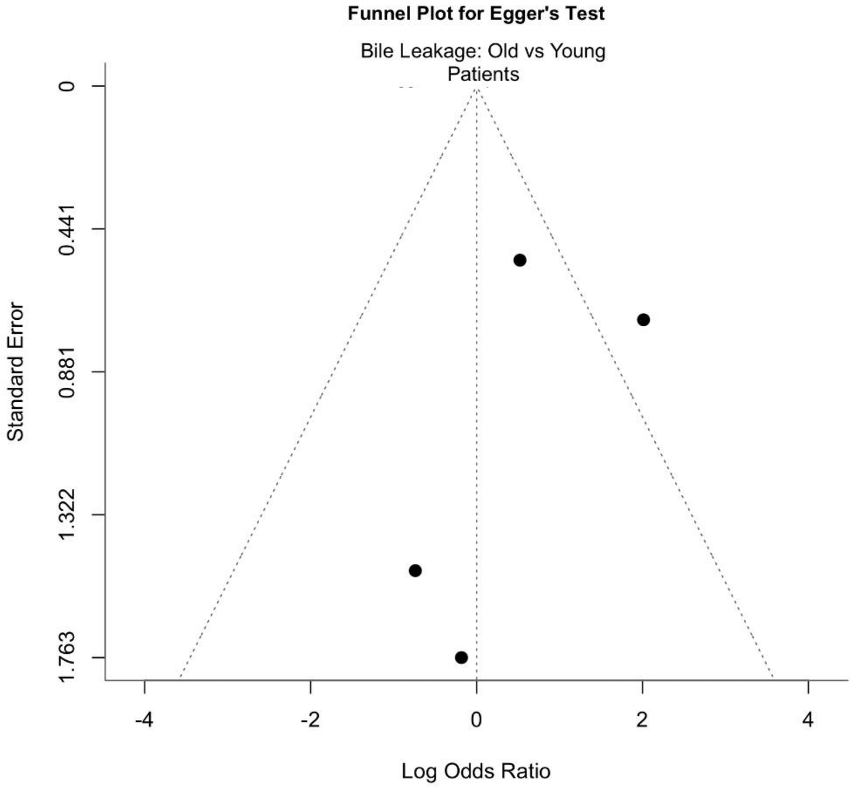

Figure S13: Funnel plot for hemorrhage

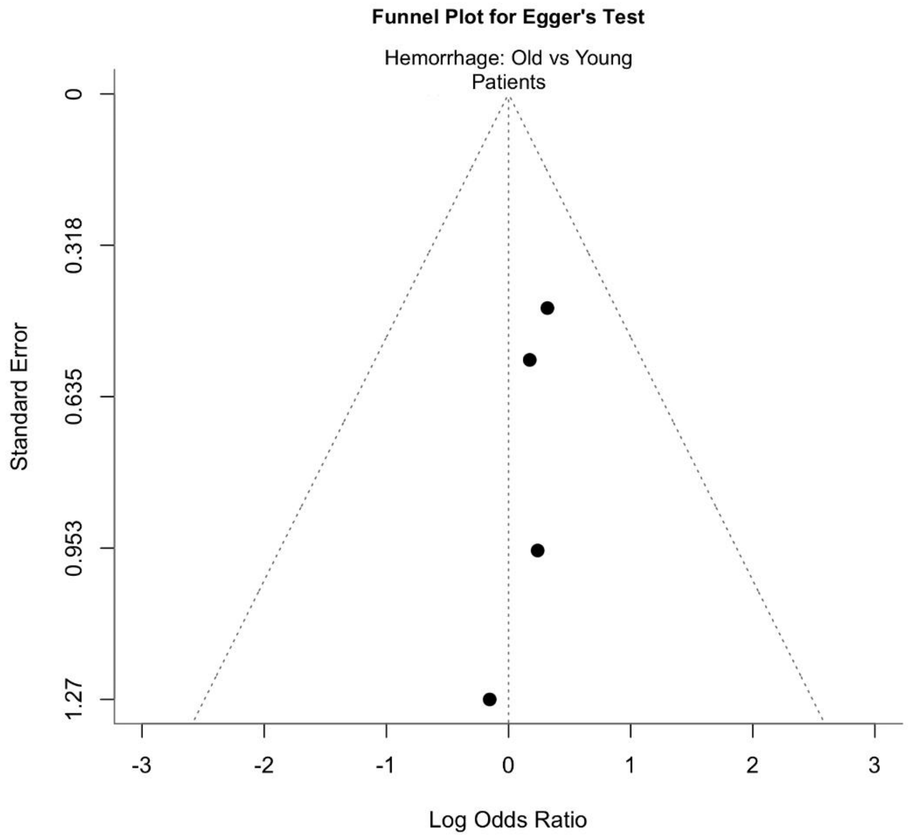

Figure S14: Funnel plot for readmission

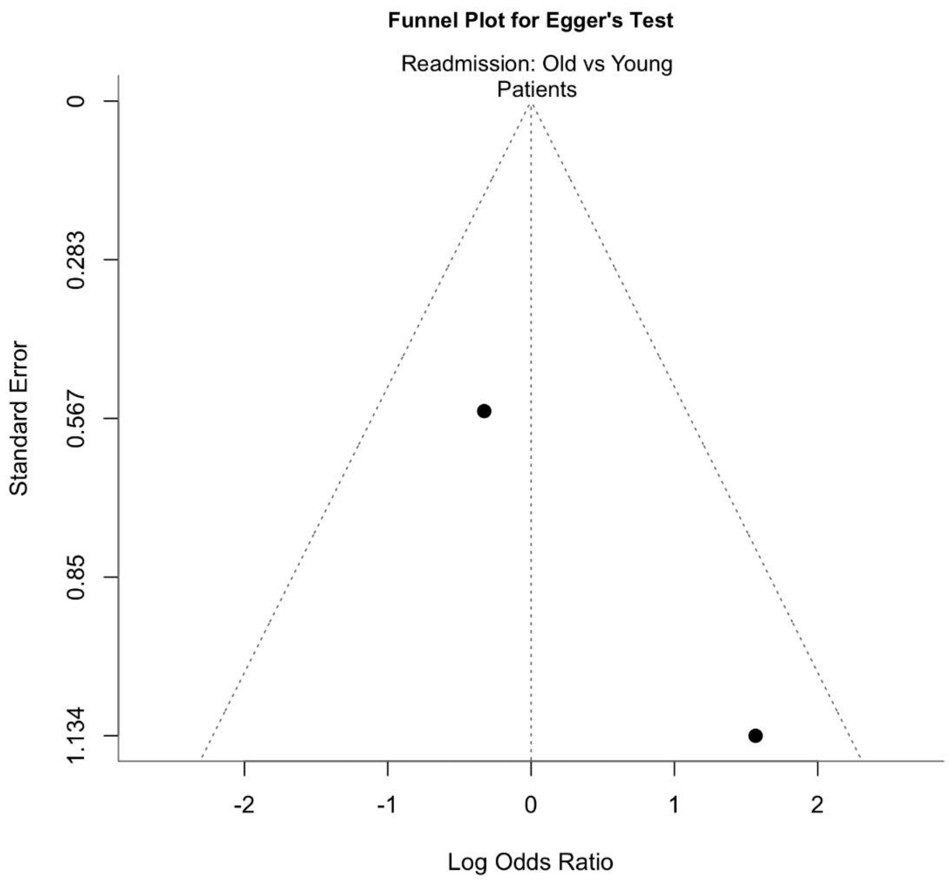

Supplement: Supplementary file 1 [file jcm-15-02744-s001.zip › Supplementary File S2.pdf]
