# Supplementary material for: Robotic Pancreaticoduodenectomy in Elderly vs. Younger Patients: Systematic Review with Meta-Analysis
Source: J Clin Med. 2026 Apr 5;15(7):2744. doi: 10.3390/jcm15072744 (PMC13072716; doi:10.3390/jcm15072744)
Supplement: Supplementary file 1 [file jcm-15-02744-s001.zip › Supplementary Tables S1-S3.pdf]

Table S1: Risk of Bias Assessment based on the Newcastle-Ottawa Scale

Studies	Selection	Comparability	Outcome	Overall
Liu	4/4	2/2	3/3	Good
Takagi	4/4	2/2	3/3	Good
Buchs	3/4	1/2	3/3	Good
Shyr	4/4	2/2	3/3	Good
Paolini	4/4	2/2	3/3	Good

Table S2: Age cutoff subgroup analysis for OR effect size

Outcomes	Age cutoff at 70 (OR)	Age cutoff at 80 (OR)	p-value
Transfusions	1.41	3.76	0.0832
Complications	1.95	0.88	0.0091
Mortality	2.39	4.69	0.0223
Clavien-Dindo III-IV	2.44	2.00	0.0265
Infection	1.25	1.08	0.8445
Reoperation	1.65	1.43	0.7121
Readmission	-	-	-
Bile Leakage	1.45	3.29	0.4135
Clinical Pancreatic Fistula	1.48	1.32	0.3433
Hemorrhage	1.30	1.19	0.7587
Delayed Gastric Emptying	1.49	4.33	<0.0001

Table S3: Age cutoff subgroup analysis for MD effect size

Outcomes	Age cutoff at 70 (MD)	Age cutoff at 80 (MD)	p-value
Operative Time	5.3	27.4	0.2446
Blood Loss	35.4	59.9	0.1275
Length of Stay	8.66	6.68	<0.0001
